# Anakinra, an interleukin-1 receptor antagonist, increases the morphine analgesic effect and decreases morphine tolerance development by modulating oxidative stress and endoplasmic reticulum stress in rats

**DOI:** 10.3906/sag-2005-256

**Published:** 2020-12-17

**Authors:** Onur AVCI, Ahmet Şevki TAŞKIRAN

**Affiliations:** 1 Department of Anesthesiology and Reanimation, Faculty of Medicine, Sivas Cumhuriyet University, Sivas Turkey; 2 Department of Physiology, Faculty of Medicine, Sivas Cumhuriyet University, Sivas Turkey

**Keywords:** Anakinra, morphine analgesia, morphine tolerance, oxidative stress, endoplasmic reticulum stress

## Abstract

**Background/aim:**

Recent studies have shown that inflammation plays a role in morphine analgesia and tolerance development. Anakinra is a competitive inhibitor of IL-1 receptors and an antiinflammatory protein regulating IL-1β’s biological activity by avoiding signal transduction. In this study, we aimed to examine the effects of anakinra on morphine analgesia and tolerance.

**Materials and methods:**

In this study, 36 Wistar Albino (230–250 g) male rats were used. Animals were divided into 6 groups: saline (S), 100 mg/kg anakinra (A), 5mg/kg morphine (M), M+A, morphine tolerance (MT), and MT+A. The resulting analgesic effect was measured with hot plate and tail-flick analgesia tests. After the analgesia tests, the dorsal root ganglions (DRG) tissues were removed. Oxidative stress parameters [total antioxidant status (TAS), total oxidant status (TOS)], endoplasmic reticulum (ER) stress, and apoptosis proteins [E74-like factor 2 (elF-2α), activating transcription factor 4 (ATF-4), C/EBP homologous protein (CHOP), caspase-3, and bcl-2-associated X protein (bax)] were measured in DRG tissues.

**Results:**

Anakinra showed an antinociceptive effect when given alone (P < 0.001). In addition, anakinra increased the analgesic effect of morphine (P < 0.05 to P < 0.001), and also decreased the tolerance to morphine at a significant level (P < 0.05 to P < 0.001). Moreover, it decreased oxidative stress and ER-stress when given as a single-dose morphine and tolerance induction (P < 0.01 to P < 0.001). Furthermore, anakinra decreased apoptosis proteins after tolerance development (P < 0.001).

**Conclusion:**

Anakinra has antinociceptive properties, and it increases the analgesic effect of morphine and also prevents tolerance development. These effects probably occur by the modulation of oxidative stress and ER-stress pathways.

## 1. Introduction

Morphine is an opiate receptor agonist and analgesic that is routinely administered in cases of strong and chronic pain in clinics. The duration of morphine’s effect is reduced by the development of tolerance to its antinociceptive properties. Despite the many studies that have been made examining the development of opioid tolerance, it is still unclear as to what exactly causes this. Recent studies propose that an endogenous proinflammatory cytokine, especially interleukin 1 beta (IL-1 β), linked to the regulation of many physiological and pathological pathways, is involved in morphine analgesia and tolerance development [1–3].

Glia cells have important roles in facilitating pain by secreting proinflammatory cytokines, such as IL-1 [4]. However, exogenous IL-1 administration into periphery, brain, or spinal cord causes hyperalgesia [5]. Systemic or intrathecal IL-1 neutralizing antibodies treatment or IL-1ra treatment decrease or block the hyperalgesia caused by some inflammatory stimuli [6, 7]. It has been reported that the deletion of IL-1 receptor types I or of the IL-1 receptor accessory protein and transgenic overexpression of IL-1 receptor antagonists in the brain and spinal cord, and in normal mice treated chronically with IL-1ra, show low pain sensitivity at significant levels [8]. Based on these findings, it is possible to argue that IL-1 signaling modulates the sensitivity of pain in basal and inflammatory conditions.

Anakinra is the recombinant form of IL-1ra and is the first biologic agent designed to modify the biological immune response of IL-1. Previous studies reported that it improves the clinical markers of rheumatoid arthritis (RA) at significant levels. It was approved by the Food and Drug Administration (FDA) in 2001 for patients who had moderate-severe RA [9]. Moreover, anakinra currently is used for Covid-19 treatment in intensive care units [10]. However, it has been found that anakinra has antinociceptive effects on the paclitaxel-induced neuropathic pain model [11].

Several studies of IL-1 receptor blockers using various experimental nociceptive models have shown that they have antinociceptive properties [12,13]. However, the effect of anakinra on acute nociception, morphine analgesia, and tolerance development are still unclear. The purpose of the current study was to examine the possible involvement of anakinra on nociception, morphine analgesia, and morphine tolerance development involving oxidative stress and ER-stress pathways in rats.

## 2. Materials and methods

### 2.1. Animals

Wistar Albino rats (230–250 g; n = 6 for each group; in total 36 rats were used) were acquired from the Animal Center Laboratory of Cumhuriyet University (Sivas, Turkey), and kept in standard conditions: a 12-h light and dark cycle (lights turned on at 08:00 AM) with ad libitum food and water at constant temperature (22 ± 2 °C). All of the experiments were performed between 09:00 and 17:00. The animals were handled and the procedures were carried out in accordance with the National Institute of Health’s guidelines: “Principles of Animal Laboratory Care.” The Sivas Cumhuriyet University Animal Ethics Committee approved the experimental protocols (Approval No.: 65202830-050.04.04-356).

### 2.2. Drugs

Morphine sulfate (Sivas Cumhuriyet University Hospital, Sivas, Turkey) and anakinra (Kineret, 100 mg/0.67 mL ampule) were dissolved in saline solution. The drugs were freshly dissolved on trial days. Morphine (5 mg/kg) was administered subcutaneously (s.c.) and anakinra (100 mg/kg) intraperitoneally (i.p.) before analgesia tests.

### 2.3. Experimental protocols

**Figure 1:**
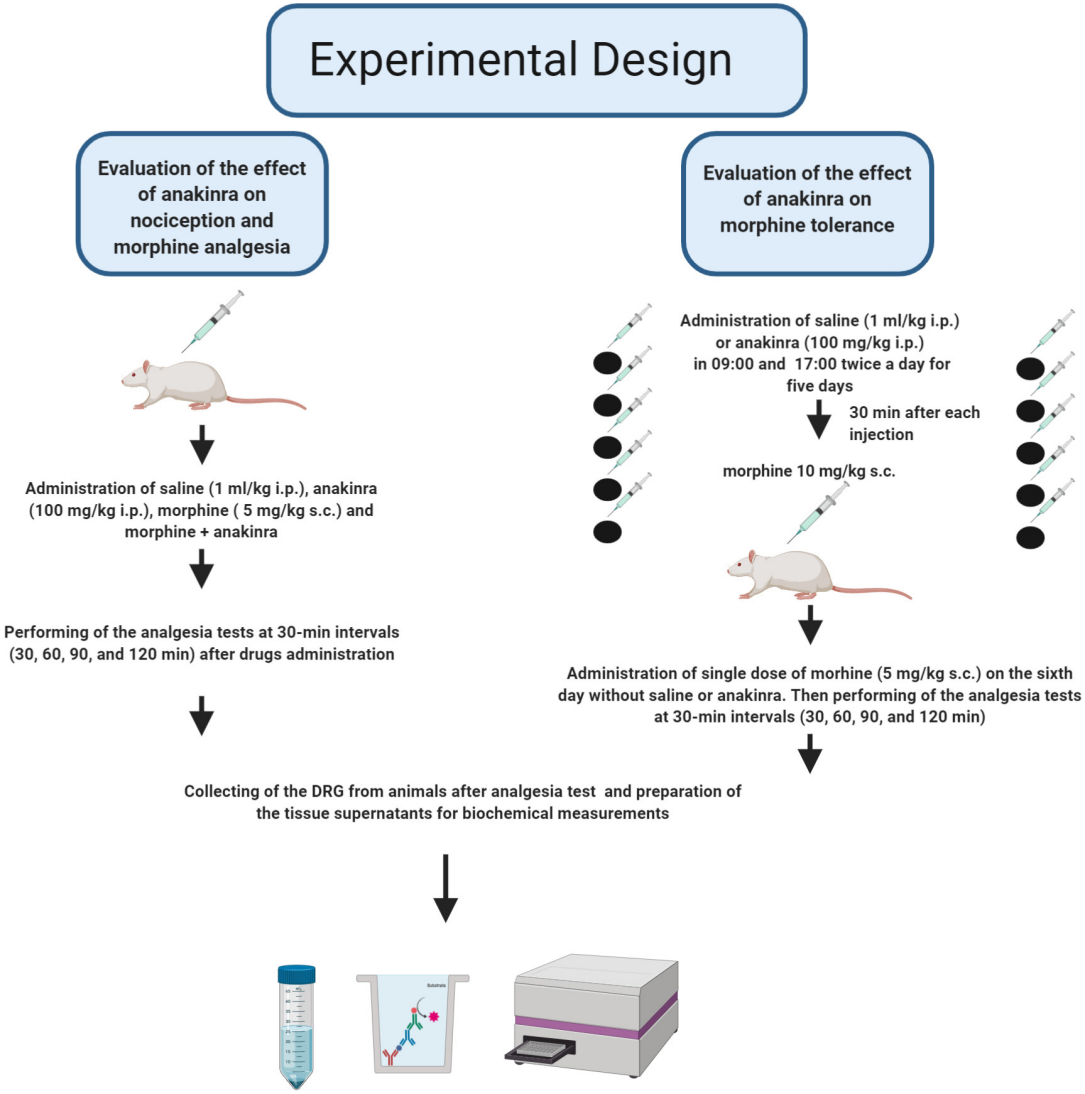
Experimental design of the study.

Anakinra and morphine’s analgesic effects were evaluated at 30-min intervals (30, 60, 90, and 120 min) using tail-flick and hot-plate antinociception tests. The animals were separated into 6 groups: saline (S), 100 mg/kg anakinra (A), 5mg/kg morphine (M), M+A, morphine tolerance (MT), and MT+A. Saline and anakinra were administered i.p. and morphine was administered s.c. at the indicated doses (volume of administration, 1 mL/kg). After analgesic tests, the animals were sacrificed by decapitation. The dorsal root ganglions (DRG) tissue (T12-L5 levels) obtained from the animals underwent assessments (Figure 1).

### 2.4. Antinociception tests

**Tail-flick test**

A standard tail-flick device (May TF 0703 Tail-flick Unit, Commat, Turkey) was used to measure thermal nociception. The radiant heat source was focused on the distal portion of the tail at a distance of 3 cm in each measurement after the administration of saline or the study’s drugs. Tail-flick latencies (TFL) were measured once the saline or the drugs had been administered. The cut-off latency time was adjusted to 15 s to prevent tissue injury. The hyperalgesic response in the tail-withdrawal test is associated with central pain mechanisms.

**Hot-plate test**

The antinociceptive reaction on the hot-plates is thought to stem from central and peripheral mechanisms together. Animals were placed one-by-one on a hot-plate (May AHP 0603 Analgesic Hot-plate, Commat, Turkey) at 54 ± 3 °C. The lag until the first paw-licking or jump reaction to avoid heat was recorded as a pain threshold indicator. The cut-off time was 30 s to prevent damage to paws.

### 2.5. Morphine tolerance induction

To induce morphine tolerance, the rats were selected at random and treated s.c. with 10 mg/kg morphine twice a day (09:00 and 17:00) for 5 days. Furthermore, morphine (10 mg/kg) was applied for 30 min after each anakinra injection for 5 days in order to determine the impacts of anakinra (100 mg/kg, i.p.) on morphine tolerance. The optimal analgesic morphine dose (5 mg/kg, s.c.) was given on the 6th day without saline or anakinra, and the tail-flick and hot-plate tests were measured at 30-min intervals (30, 60, 90, and 120 min) in order to evaluate the degree of tolerance.

### 2.6. DRG tissue homogenate preparation

DRG tissue samples of the animals in cold phosphate-buffer saline solution were homogenized using a mechanical homogenizer (Analytik Jena Speed Mill Plus, Jena, Germany) and then centrifuged at 4000 rpm for 10 min at a temperature of 4 ºC. Then, the supernatants were obtained and stored at –80 °C until biochemical analysis. A Bradford protein assay kit (Merck, Germany) was used to determine total protein levels in the samples [14].

### 2.7. Total antioxidant status (TAS) measurement

TAS concentrations at tissue level were determined with an automated assay method that was previously developed by Erel [15] which was based on monitoring the reaction rate of free radicals by measuring the absorbance of colored dianisidyl radicals during free radical reactions starting with hydroxyl radical production in a Fenton reaction. Antioxidants in the tissue samples should suppress coloring proportionally to their concentrations [15]. The outcomes were expressed in micromolar Trolox equivalents per gram tissue protein (μmol Trolox Eq/g protein).

### 2.8. Total oxidant status (TOS) measurement

Tissue TOS concentrations were quantified with Erel’s automated assay method [16]. Because ferrous ion is oxidized to ferric ion when adequate quantities of oxidants are available in the medium, the method allows for quantifying TOS levels by measuring the tissue levels of ferric ions with the use of xylenol orange. Hydrogen peroxide was used for the calibration of the assay [16]. The results of the assay were expressed in micromolar hydrogen peroxide equivalents per gram tissue protein (μmol H2O2 Eq/g protein).

### 2.9. Measurement of elF-2α, ATF-4, CHOP, caspase-3, and bax

The levels of elF-2α, ATF-4, CHOP, caspase-3, and bax from DRG supernatants were measured using rat ELISA commercial kits (Shanghai Sunred Biological Technology, Shanghai, China). Operation protocols were in line with the instructions of the manufacturer. In brief, standard and tissue samples were added in a plate and incubated for 60 min at 37 °C. After washing, staining solutions were added and incubated for 15 min at 37 °C. Stop solution was added and read at 450 nm. Standard curves were employed in calculating all kits. The variation coefficients in and between plates were lower than 10%.

### 2.10. Analgesic tests data analysis

To calculate the maximum antinociceptive effect percentage (% MPE), tail-flicks and hot-plate lags (which are in seconds) were converted into an antinociceptive effectiveness percentage with the following equation: % MPE = [(post drug latency – baseline latency) / (cut-off value – Baseline latency)] × 100.

### 2.11. Statistical analysis

The results are given as mean ± SEM (standard error of the mean). The antinociceptive effect was measured, and the mean % MPEs were calculated. Analysis of variance (One-Way Anova) and a posthoc Tukey test were used in analyzing the data. The significance value was set as P < 0.05.

## 3. Results

### 3.1. Effect of anakinra on nociception and morphine analgesia

**Figure 2:**
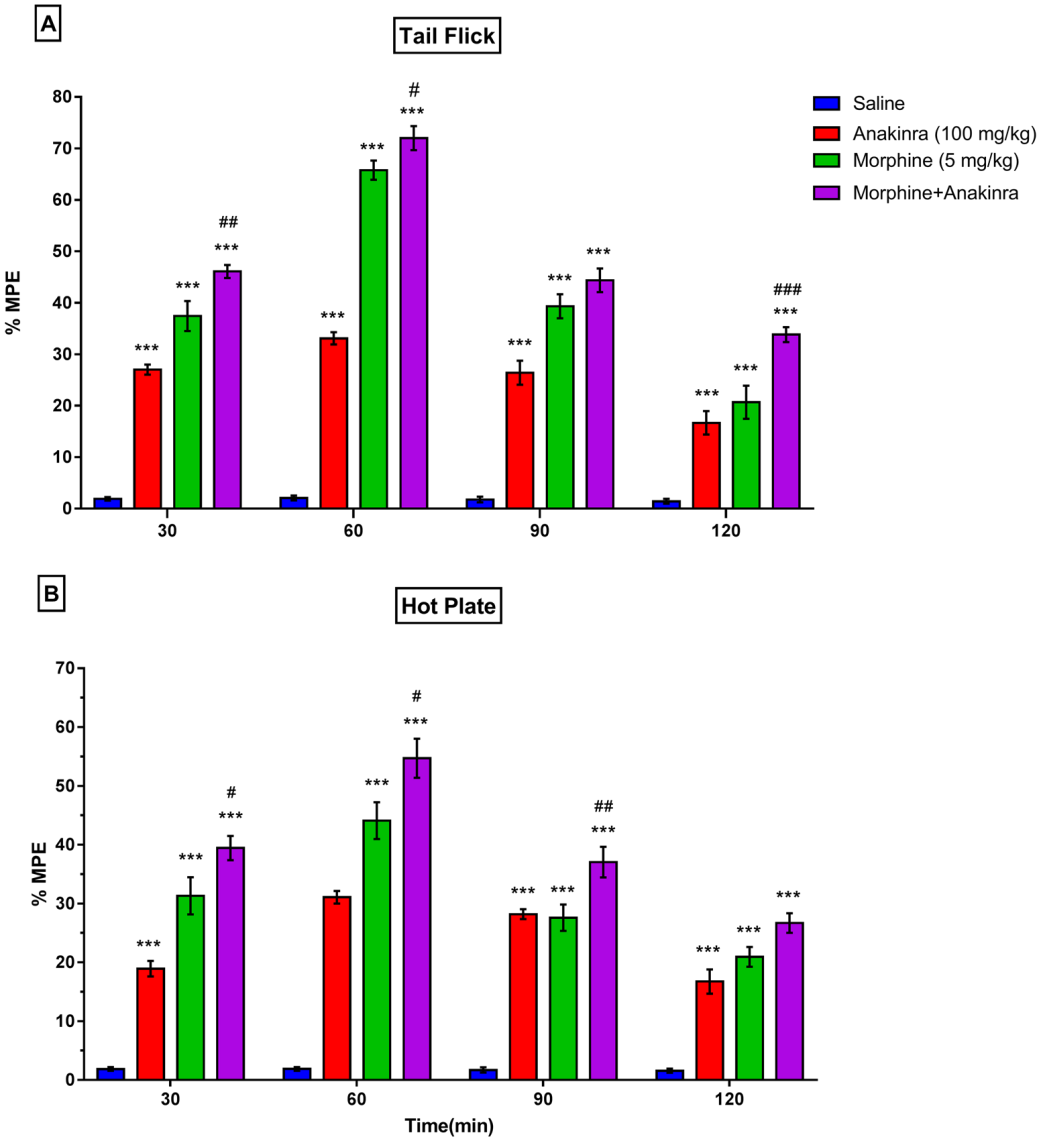
Effect of anakinra on nociception and morphine analgesia. (a) shows the effect of anakinra on nociception and morphine analgesia in the tail flick test; (b) shows the effect of anakinra on nociception and morphine analgesia in the hot plate test. Values are expressed as the means ± SEM of % MPE (n = 6). ***P < 0.001 compared to saline group. #P < 0.05, ##P < 0.01, and ###P < 0.001, compared to the morphine group.

In order to examine anakinra, analgesic responses were evaluated for a 100 mg/kg dose of anakinra at 30 min intervals for 2 h with the analgesia tests. Anakinra showed antinociceptive effects in comparison with the saline group at 30, 60, 90, and 120 m in both the tail-flick test (P < 0.001; Figure 2a) and hot plate test (P < 0.001; Figure 2b). However, the maximum analgesic effect was found at 60 min after 100-mg/kg anakinra administration for the tail-flick (33.08 ± 1.17) and hot-plate test (31.06 ± 1.06).

The findings demonstrated that anakinra significantly increased the antinociceptive effect of morphine in the tail-flick test (P < 0.05 to P < 0.001; Figure 2a) and hot plate test (P < 0.05 to P < 0.01; Figure 2b) in comparison with the morphine group. Furthermore, the maximum increasing effect of anakinra on morphine was detected at 60 min in the tail-flick test (71.99 ± 2.34) and hot-plate test (54.70 ± 3.33).

### 3.2. Effect of anakinra on morphine tolerance development

**Figure 3:**
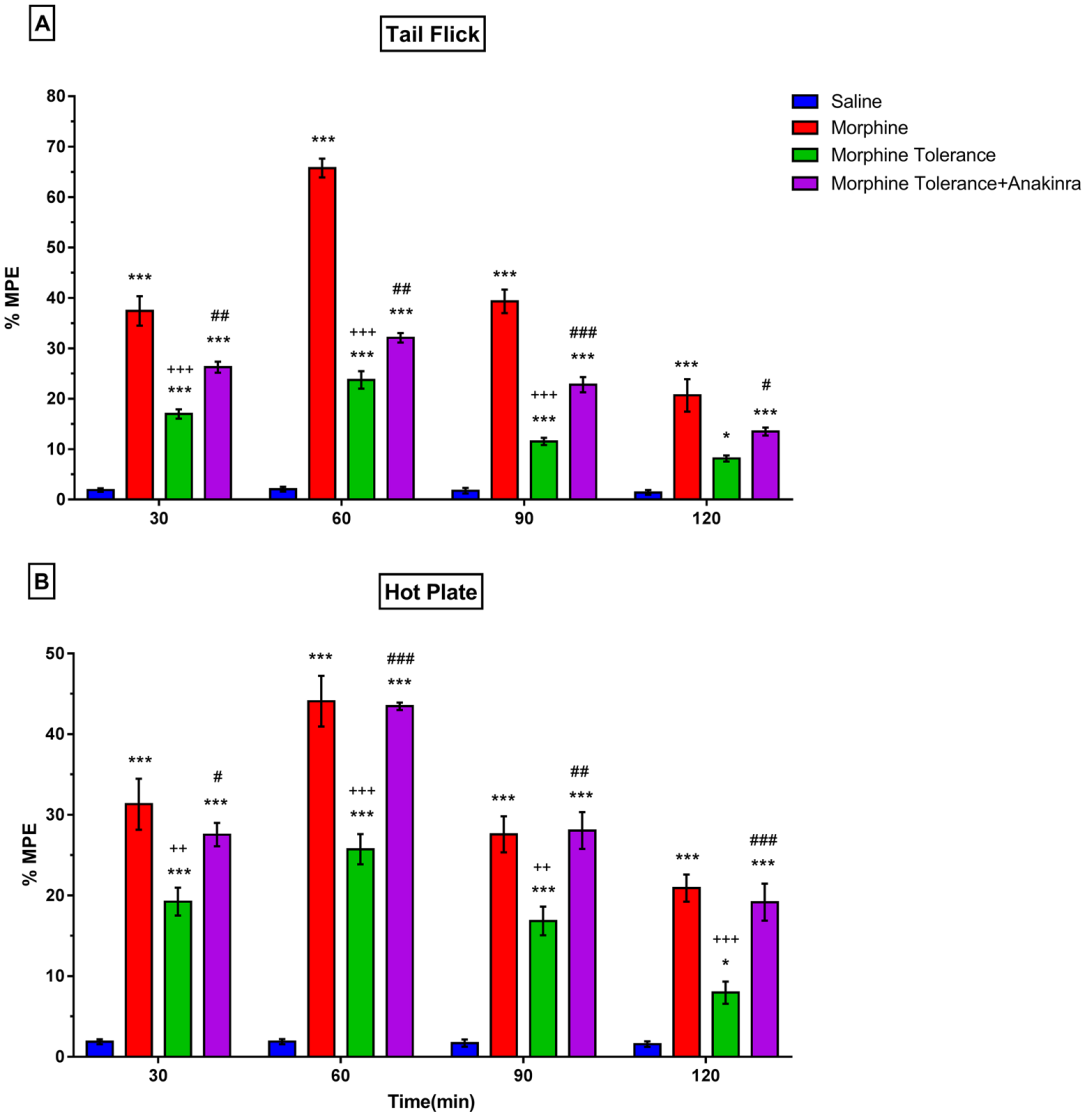
Effect of anakinra on morphine tolerance development. (a) shows the effect of anakinra on morphine tolerance development in the tail flick test; (b) shows the effect of anakinra on morphine tolerance development in the hot plate test. Values are expressed as the means ± SEM of % MPE (n = 6). *P < 0.05 and ***P < 0.001, compared to the saline group. ++P < 0.01 and +++P < 0.001, compared to morphine group. #P < 0.05, ##P < 0.01, and ###P < 0.001, compared to the morphine tolerance group.

The morphine group’s % MPE value was statistically higher than the morphine tolerance group in both the tail-flick test (P < 0.05 to P < 0.001; Figure 3a) and hot plate test (P < 0.05 to P < 0.001; Figure 3b). Anakinra with morphine produced a significantly decreased morphine tolerance development in the tail-flick test (P < 0.05 to P < 0.001; Figure 3a) and hot plate test (P < 0.05 to P < 0.001; Figure 3b).

### 3.3. Effect of anakinra on antioxidant and oxidant parameters (TAS and TOS levels) in morphine analgesia and tolerance in DRG

**Figure 4:**
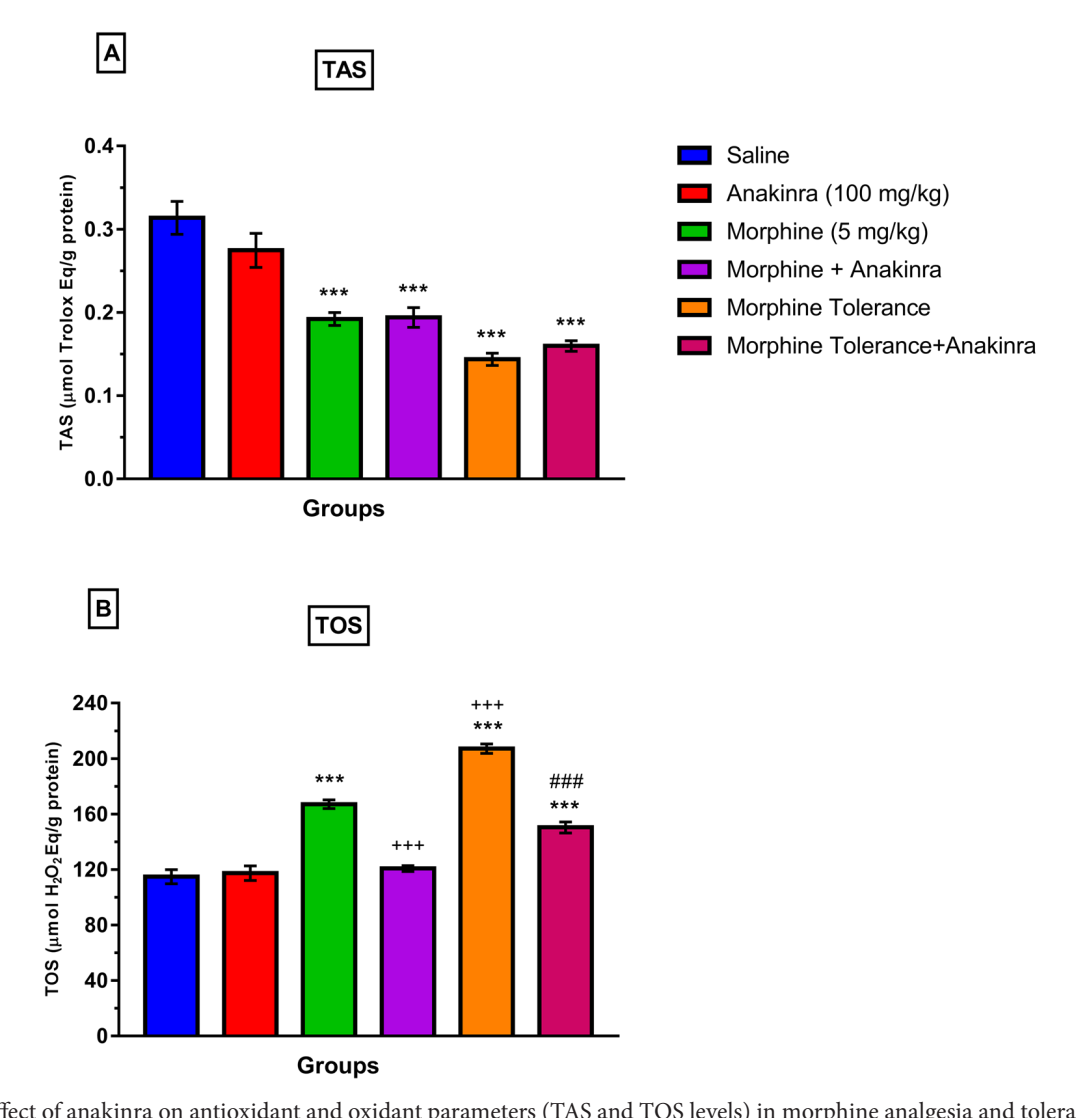
Effect of anakinra on antioxidant and oxidant parameters (TAS and TOS levels) in morphine analgesia and tolerance in DRG (a) shows the effect of anakinra on TAS levels in morphine analgesia and tolerance in DRG; (b) shows the effect of anakinra on TOS levels in morphine analgesia and tolerance in DRG. Values are expressed as the means ± SEM of % MPE (n = 6). ***P < 0.001, compared to the saline group. +++P < 0.001, compared to the morphine group. ###P < 0.001, compared to the morphine tolerance group.

Single-dose morphine administration and tolerance induction decreased significantly the TAS levels in DRG compared to saline (P < 0.001; Figure 4a). However, anakinra did not ameliorate the effects of morphine and tolerance (P > 0.05; Figure 4a), and single-dose morphine increased TOS levels in DRG compared to saline (P < 0.001; Figure 4b). Nevertheless, anakinra reduced TOS levels in DRG when combined with morphine compared to single morphine (P < 0.001; Figure 4b). Moreover, morphine tolerance increased TOS levels in DRG compared to both the saline (P <0.001; Figure 4b) and morphine group (P < 0.001; Figure 4b). In the end, anakinra reduced TOS levels in DRG together with tolerance induction compared to the morphine tolerance group (P < 0.001; Figure 4b).

### 3.4. Effect of anakinra on ER-stress proteins (elF-2α, ATF-4 and CHOP levels) in morphine analgesia and tolerance in DRG

**Figure 5:**
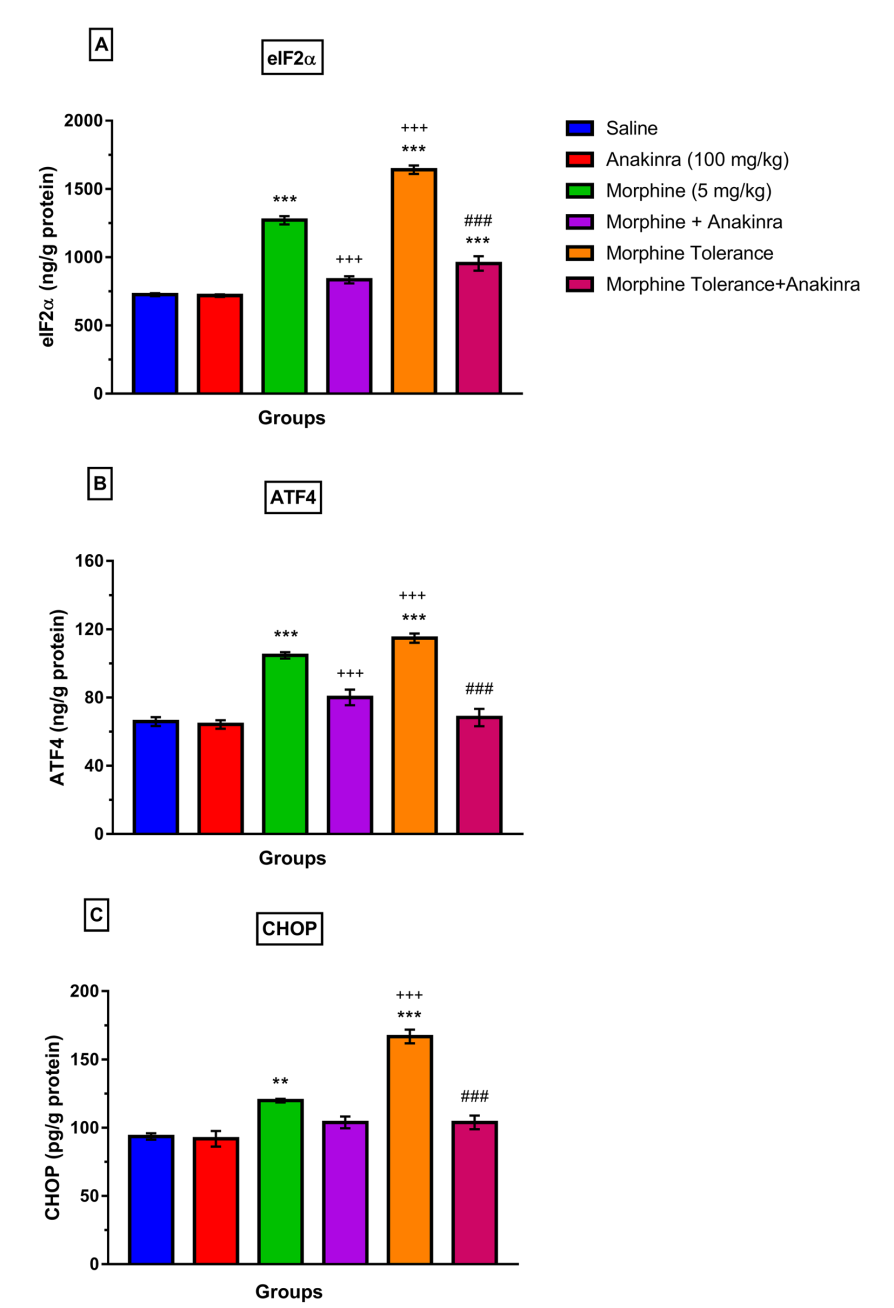
Effect of anakinra on ER-stress proteins (elF-2α, ATF-4, and CHOP levels) in morphine analgesia and tolerance in DRG (a) shows the effect of anakinra on elF-2α levels in morphine analgesia and tolerance in DRG; (b) shows the effect of anakinra on ATF-4 levels in morphine analgesia and tolerance in DRG; (c) shows the effect of anakinra on CHOP levels in morphine analgesia and tolerance in DRG. Values are expressed as the means ± SEM of % MPE (n = 6). **P < 0.01 and ***P < 0.001, compared to the saline group. +++P < 0.001, compared to the morphine group. ###P < 0.001, compared to the morphine tolerance group.

The single-dose morphine raised elF-2α levels in DRG compared to saline (P < 0.001; Figure 5a). However, anakinra reduced elF-2α levels in DRG when combined with morphine compared to single morphine (P < 0.001; Figure 5a). In addition, morphine tolerance increased elF-2α levels in DRG compared to both the saline (P < 0.001; Figure 5a) and single morphine (P < 0.001; Figure 5a). However, anakinra reduced elF-2α levels in DRG together with tolerance induction compared to the morphine tolerance group (P < 0.001; Figure 5a).

Single morphine increased ATF-4 levels in DRG compared with saline (P < 0.001; Figure 5b). However, anakinra decreased ATF-4 levels in DRG combined with morphine compared to single-dose morphine (P < 0.001; Figure 5b). Furthermore, morphine tolerance raised ATF-4 levels in DRG compared to saline (P < 0.001; Figure 5b) and single-dose morphine administration (P < 0.001; Figure 5b). Nevertheless, anakinra reduced ATF-4 levels in DRG when given together with tolerance development compared to the morphine tolerance group (P < 0.001; Figure 5b).

Single-dose morphine raised CHOP levels in DRG compared to saline (P < 0.01; Figure 5c). Although anakinra combined with morphine reduced CHOP levels in DRG, this was not at a significant level (P > 0.05; Figure 5c). Also, morphine tolerance increased CHOP levels in DRG compared to both saline (P < 0.001; Figure 5c) and single morphine (P < 0.001; Figure 5c). However, anakinra reduced CHOP levels in DRG together with tolerance induction compared to the morphine tolerance group (P < 0.001; Figure 5c).

### 3.5. Effect of anakinra on apoptosis (caspase-3 and bax levels) in morphine analgesia and tolerance in DRG

**Figure 6:**
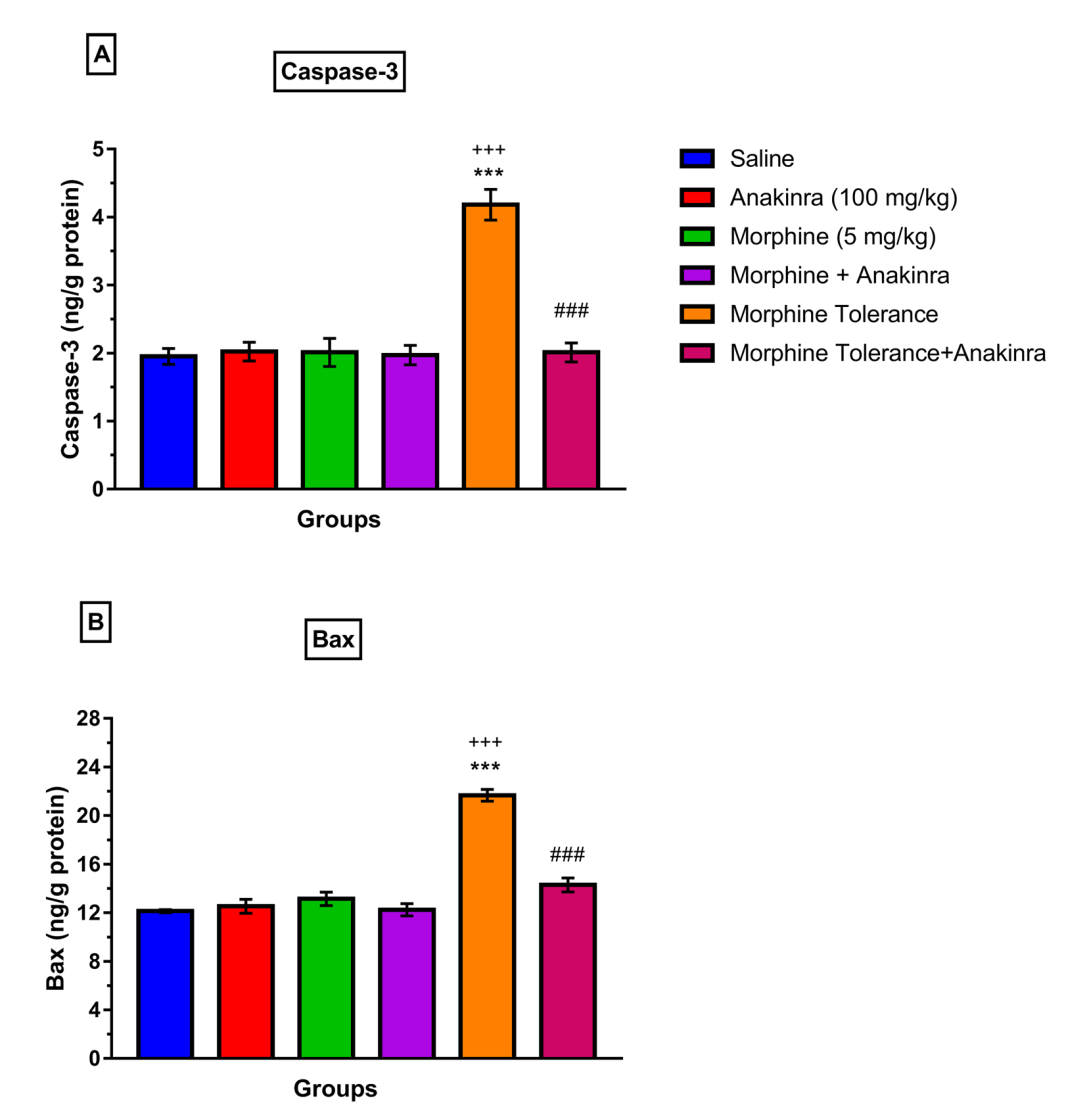
Effect of anakinra on apoptosis (caspase-3 and bax levels) in morphine analgesia and tolerance in DRG. (a) effect of anakinra on caspase-3 levels in morphine analgesia and tolerance in DRG; (b) shows the effect of anakinra on bax levels in morphine analgesia and tolerance in DRG. Values are expressed as the means ± SEM of % MPE (n = 6). ***P < 0.001, compared to the saline group. +++P < 0.001, compared to the morphine group. ###P < 0.001, compared to the morphine tolerance group.

Single morphine administration did not change caspase-3 levels in DRG compared to saline (P > 0.05; Figure 6a). However, morphine tolerance raised caspase-3 levels in DRG compared to saline (P < 0.001; Figure 6a) and also single-dose morphine administration (P < 0.001; Figure 6a). Furthermore, anakinra reduced caspase-3 levels in DRG when given together with tolerance development compared to the morphine tolerance group (P < 0.001; Figure 6a).

Single-dose morphine did not also alter bax levels in DRG compared to saline (P > 0.05; Figure 6b). Nevertheless, morphine tolerance increased bax levels in DRG compared to both the saline (P < 0.001; Figure 6b) and single morphine groups (P < 0.001; Figure 6b). On the other hand, anakinra reduced bax levels in DRG given together with tolerance induction compared to the morphine tolerance group (P < 0.001; Figure 6b).

## 4. Discussion

In this study, we investigated the impacts of anakinra on nociception, morphine analgesia, tolerance development, and possible mechanisms. An IL-1 receptor antagonist, anakinra showed a marked antinociceptive effect, enhanced morphine analgesia, and also decreased morphine tolerance development. The results are in line with previous studies reporting that blockage of the IL-1 receptor has an antinociceptive effect and increases morphine analgesia and tolerance development [1,11]. Moreover, some previous studies have suggested that mice strains characterized with high endogenous IL-1 (beige-J mutation and diabetic mice) levels showed resistance to morphine analgesia. These results are in line with the report of another study arguing that an intracerebroventricular-neutral dose of IL-1b administration eliminated morphine’s analgesic effect in diabetic and control mice [17]. Furthermore, some studies have shown morphine-induced IL-1 production. Chronic in vivo morphine administration to nerve-transected and control rats activated spinal glia, upregulated spinal mRNA, and protein levels of proinflammatory cytokines (especially IL-1b and IL-6) [18]. Chronic morphine also improved IL-1’s mRNA expression in peritoneal macrophages [19]. Morphine exposure activated IL-1b synthesis by macrophages under in vitro conditions [20]. Corroborating the present findings, a study reported that intrathecal IL-1ra potentiated acute morphine analgesia [2]. Moreover, it has been suggested that the blocking of acute morphine activates satellite glial cells, upregulating IL-1b in DRG in mice with matrix metalloprotease-9 [21]. Taken together, these studies show that both chronic and acute morphine are related to an increase in proinflammatory cytokines in the nervous system, and this is one of the reasons why both types restrain morphine analgesia and trigger morphine tolerance development. Therefore, the administration of anakinra increases morphine analgesia and reduces morphine tolerance development by blocking the engagement of IL-1 cytokine to its receptor.

Systemic morphine use ended in oxidative stress with a reduction in decreased glutathione levels, in lipid peroxide malondialdehyde levels, and peroxynitrite production in continuous use, which occurred simultaneously with tolerance and dependence development. Antioxidants, such as thymoquinone and those that target peroxynitrite formation, reversed biochemical changes and morphine tolerance and dependence [22,23]. In this study, it was found that single-dose morphine and also morphine chronic administration for development tolerance decreases antioxidant status (TAS) in DRG, and this is consistent with previous studies. However, anakinra altered TAS levels neither together with single morphine nor tolerance in DRG. It may show that morphine use suppresses the antioxidant system, and this may be related to development tolerance. Furthermore, it also was also shown that single-dose morphine and chronic morphine administration cause oxidative stress (TOS) in DRG in this study. Moreover, chronic morphine administration increased TOS levels more in single-dose than in DRG. This may be associated with tolerance development. However, anakinra alleviated these morphine-related effects.

Endoplasmic reticulum stress was associated with participation in neuropathic pain mechanisms [24,25] and inflammatory pain [26]. In addition, ER stress activation was found in the peripheral nervous system of diabetic nephropathy rats [24]. Moreover, a few recent studies have reported that ER stress has a role in morphine analgesia and tolerance mechanisms [27,28]. It has also been found that PERK/eIF2a ER stress pathway activation increased after tolerance in the spinal cord [28]. In this study, it was found that ER stress proteins (elF-2α, ATF-4 and CHOP) increased in single-dose morphine and chronic morphine administration in DRG. However, chronic morphine administration increased ER stress proteins more than single-dose morphine in DRG. At the same time, anakinra reversed the effects of morphine.

Previous studies have reported that morphine tolerance is implicated in neuronal apoptosis by mediating some cellular mechanisms such as oxidative stress and ER stress [29,30]. Our findings have indicated that morphine tolerance induced apoptosis in DRG by increasing caspase-3 and bax in DRG. This is consistent with a previous study. However, the acute administration of single morphine did not activate apoptosis despite increasing oxidative stress and ER stress. It may be related to the fact that there is a threshold for apoptosis in DRG. Otherwise, anakinra decreased apoptosis when giving tolerance by decreasing caspase-3 and bax in DRG.

## 5. Conclusion

Findings from this study showed that anakinra has antinociceptive properties, increases morphine analgesic effect, and also prevents tolerance development against chronic administration of morphine, possibly through inhibition of proinflammatory IL-1 cytokine adhesion to its receptor, blocking of oxidative stress, and suppressing ER stress in DRG. Thus, anakinra might be a potential therapeutic agent in morphine tolerance development management in clinical environments, especially in intensive care units.

## Informed consent

The Sivas Cumhuriyet University Animal Ethics Committee approved the experimental protocols for this study (Approval No.: 65202830-050.04.04-356).

## References

[ref1] (2005). Interleukin-1 antagonizes morphine analgesia and underlies morphine tolerance. Pain.

[ref2] (2004). A role for proinflammatory cytokines and fractalkine in analgesia, tolerance, and subsequent pain facilitation induced by chronic intrathecal morphine. Journal of Neuroscience.

[ref3] (2008). Proinflammatory cytokines oppose opioid-induced acute and chronic analgesia. Brain Behaviour Immunity.

[ref4] (2000). The pain of being sick: implications of immune-to-brain communication for understanding pain. Annual Review of Psychology.

[ref5] (1988). Interleukin-1β as a potent hyperalgesic agent antagonized by a tripeptide analogue. Nature.

[ref6] (2001). Interleukin-1 β-mediated induction of Cox-2 in the CNS contributes to inflammatory pain hypersensitivity. Nature.

[ref7] (2000). Cytokine-mediated inflammatory hyperalgesia limited by interleukin-1 receptor antagonist. British Journal of Pharmacology.

[ref8] (2003). Impairment of interleukin-1 (IL-1) signaling reduces basal pain sensitivity in mice: Genetic, pharmacological and developmental aspects. Pain.

[ref9] (2009). Anakinra for rheumatoid arthritis: A systematic review. The Journal of Rheumatology.

[ref10] (2020). Interleukin-1 blockade with high-dose anakinra in patients with COVID-19, acute respiratory distress syndrome, and hyperinflammation: a retrospective cohort study. The Lancet Rheumatology.

[ref11] (2016). The effect of anakinra on paclitaxel-induced peripheral neuropathic pain in rats. Turkish Journal of Anaesthesiology&Reanimation.

[ref12] (2004). Possible association of interleukin 1 gene locus polymorphisms with low back pain. Pain.

[ref13] (2005). Minocycline attenuates mechanical allodynia and proinflammatory cytokine expression in rat models of pain facilitation. Pain.

[ref14] (1994). The Bradford method for protein quantitation. Methods in Molecular Biology.

[ref15] (2004). A novel automated method to measure total antioxidant response against potent free radical reactions. Clinical Biochemistry.

[ref16] (2005). A new automated colorimetric method for measuring total oxidant status. Clinical Biochemistry.

[ref17] (2000). The interaction between IL-1β and morphine: Possible mechanism of the deficiency of morphine-induced analgesia in diabetic mice. Pain.

[ref18] (2004). DeLeo JA. Neuropsychopharmacology.

[ref19] (2000). Morphine enhances interleukin-12 and the production of other pro-inflammatory cytokines in mouse peritoneal macrophages. Journal of Leukocyte Biology.

[ref20] (2002). The immunosuppressive effects of chronic morphine treatment are partially dependent on corticosterone and mediated by the μ-opioid receptor. Journal of Leukocyte Biology.

[ref21] (2012). Acute morphine activates satellite glial cells and up-regulates IL-1β in dorsal root ganglia in mice via matrix metalloprotease-9. Molecular Pain.

[ref22] (2007). Therapeutic manipulation of peroxynitrite attenuates the development of opiate-induced antinociceptive tolerance in mice. The Journal of Clinical Investigation.

[ref23] (2013). Inhibition of brain oxidative stress and inducible nitric oxide synthase expression by thymoquinone attenuates the development of morphine tolerance and dependence in mice. European Journal of Pharmacology.

[ref24] (2015). Endoplasmic reticulum stress in the peripheral nervous system is a significant driver of neuropathic pain. Proceedings of the National Academy of Sciences of the United States of America.

[ref25] (2015). Endoplasmic reticulum stress impairment in the spinal dorsal horn of a neuropathic pain model. Scientific Reports.

[ref26] (2012). Endoplasmic reticulum stress contributes to CRH-induced hippocampal neuron apoptosis. Experimental Cell Research.

[ref27] (2014). Naloxone induces endoplasmic reticulum stress in PC12 cells. Molecular Medicine Reports.

[ref28] (2018). Endoplasmic reticulum stress in spinal cord contributes to the development of morphine tolerance. Frontiers in Molecular Neuroscience.

[ref29] (2002). Neuronal apoptosis associated with morphine tolerance: Evidence for an opioid-induced neurotoxic mechanism. Journal of Neuroscience.

[ref30] (2003). Role of heme oxygenase–1 in morphine‐modulated apoptosis and migration of macrophages. The Journal of Infectious Diseases.

